# The antidepressant effect of physical exercise: Evidence from China Family Panel Studies

**DOI:** 10.1371/journal.pone.0274321

**Published:** 2022-10-06

**Authors:** Chenchen Ren, Chao Wang, Man Zhang

**Affiliations:** 1 Capital University of Economics and Business, Beijing, China; 2 College of Business, Shanghai University of Finance and Economics, Shanghai, China; Universiti Malaya, MALAYSIA

## Abstract

Multiple studies have proved that participating in sports can effectively reduce adults’ depression. This paper provides evidence from China by using the survey data from China Family Panel Studies (CFPS), which contains sport-types, personal characteristics, and CES-D20 depression-scale score data of 33,236 individuals. In addition to the Ordinary Least Squares regression model, we adopt the Two-way Fixed Effect and Propensity Score Matching method to alleviate the endogeneity. The empirical result shows that for every additional time of physical exercise, the depression level drops by an average of 0.152; the depression level of people who participate in sports is significantly lower than that of non-participants by 0.397 points. The lowering effect of physical activity on depression is not linear, and excessive exercise may lead to increased depression. Furthermore, heterogeneity analyses discover that with the increase of age and education, the impact continued to expand. For every increase in physical exercise of the group over 76-year old, the depression level decreased by 0.373 points; while for individuals with primary school education, their depression level decreased only by 0.124 points.

## Introduction

In a fast-paced modern society, mental health has become an important factor in evaluating people’s quality of life and happiness. Data shows that the prevalence rate of mood disorder in China is 4.06%, of which the prevalence rate of depression reaches 2.1%, and the number of patients suffering from depression is about 54 million. The annual cost of absenteeism and treatment due to depression is as high as 49.4 billion RMB. The vast majority of research supports the efficacy of antidepressants. According to a 2014 FDA study, patients treated with antidepressants, on average, experienced about 50 percent less depressive symptoms than those treated with a placebo [1]. Although depression can currently be alleviated with medication, there is still a risk of relapse [2]. According to Buckman et al. (2018) [3], approximately one-third of people with depression in a nonclinical sample will have more than one episode. In clinical samples, more than 75% of people with depression had multiple episodes. In addition, many people may not receive timely medication because of financial hardship and stigma. More than 35% of adults and a staggering 60.1% of teens each year receive no treatment because of their experiences. Especially in low-income and developing countries, treatments for depression tend to have higher prices. According to a World Health Organization statistic, more than 75% of people with mental disorders in these countries are not receiving treatment. Therefore, it is crucial to seek long-term, lower-cost means of improving mental health on the basis of drug therapy.

Physical exercise is not only a physical activity but also a psychological and social activity [4]. The preventive and alleviating effects of physical exercise on mental health improvement have always been the joint focus of medical, physical, and psychological studies [5]. Recent studies have shown that participation in physical exercise can significantly improve people’s thinking, imagination, memory abilities [6], and promote brain cell response speed as well [7]. Moreover, researchers have ascertained that physical exercise can stimulate the nervous central system, have a positive effect on people’s emotions [8] and have anti-depressant effects. In addition, confrontation and cooperation during the physical exercise also help residents to build up communication, go through happiness [9], and make strides in stress-bearing ability [10].

Previous research on this topic was mainly carried out through randomized controlled trials (RCT). Such a method is mainly used for reference in the medical field to test the clinical effects of certain drugs or treatment measures, which is reasonable to some extent. However, there are two drawbacks associated with the use of RCT in social science. The first disadvantage is its reliance on sample selection. Existing research often selects samples in a certain region or several regions because large-scale sampling cannot be realized under multiple restrictions. Therefore, it is difficult to have a comprehensive understanding of the problem and to compare the differences in physical exercise effects between individuals of different age groups, regions, and personal characteristics. The second limitation of RCT is medical ethics issues. Current studies often set up experimental groups and control groups to give different levels of physical exercise, and then observe the differences in experimenters’ mental health. This method has a certain degree of objectivity, but it is likely to cause a long-term psychological impact on the participants.

Therefore, expanding the representativeness of sample selection and exploring new research methods have become two important directions on this problem. Fortunately, the modern quantitative analysis provides ideas for our new research methods. In fact, in the recent ten years of related research, a few scholars, such as Baker et al. (2005) [11], Birkeland et al. (2009) [12], Rasciute and Downward (2010) [13], Win et al. (2011) [14], Waldman et al. (2012) [15] have all used large-scale micro-survey data and quantitative methods to analyze the relationship between physical exercise and depression. Chinese scholars, such as Wang et al.(2003) [16], Qiu(2004) [4], Xu et al.(2002) [17], Song(2008) [18], Sheng et al.(2016) [19], also keenly aware of the deficiencies of the controlled experiment, they start to use regional cross-sectional survey data in China and practice quantitive analysis by software. Their approach has been recognized and promoted by academic peers. However, the results are substantially affected by the regional samples and this paper provides evidence at the national level in the first time. Our research strongly support the view that doing sports significantly reduce depression symptoms. Except for sample selection, reverse causality is another concern for endogeneity. Classical researches done by Waldman et al. (2012) find that there are endogenous problems between physical exercise and mental health [15]. That is to say, although physical exercise can improve mental health, there is another possibility that mentally healthier groups prefer to choose physical exercise. If directly regress depression indicator on physical exercise, the estimated coefficient is meant to be biased and inefficient.

The China Family Panel Studies (CFPS) is conducted by the Chinese Social Science Survey Center of Peking University every two years since 2010. Survey participants cover 16,000 family members in 25 provinces, municipalities, and autonomous regions across the country. Survey questionnaire contains individual social, economic, demographic, education and health information. We use the 2016 China Family Panel Studies (CFPS) open data and modern econometric methods to make a new exploration on the question "Does physical exercise reduce the level of individual depression?" We attempt to answer this question more comprehensively and accurately from new data sources, in order to provide better theoretical support and policy guidance for the nationwide fitness strategy.

The rest of the paper is organized as follows. Section 2 reviews the related literature. Section 3 describes the data and empirical model. Section 4 documents the regression results, as well as endogenous treatment, robustness check and heterogeneity exams. Section 5 concludes and makes some extensions.

## Review of literature

In the past ten years, the medical and sports science community have conducted a lot of analysis on the relationship between physical exercise and mental health. According to the research themes, we summarize related articles into two categories: first, a general discussion about the relationship between physical exercise and mental health; second, a specific discussion about physical exercise and depression.

Research on physical exercise and mental health mainly uses questionnaire survey methods to measure physical activity and mental health. For example, Wang et al.(2003) analyzed the EPQ and CAS scientific scales of 965 university students in China and found that students who worked hard to participate in physical exercise showed better mental health [16], while students who did not work hard to participate showed more restless and anxious. Xu et al.(2002) further expanded the sample size [17], selecting mental health scale and physical exercise survey data of 386 college students and found that physical exercise contributes to 52.5% of mental health. Song(2008) used the SCI-90 scale and the survey data conducted on 8,499 undergraduates [18], found that with the increase of activity time, the proportion of undergraduates with psychological problems Gradually decreases. After the number of activities per week reaches more than 3 times, this proportion is significantly reduced. Recent studies such as Sheng et al.(2016) used SCI-90, GSES and the revised PARS-3 scale to conduct a sample survey of 1084 high school students [19]. He found that the form, content and time of physical exercise have different effects on the mental health of students, and the effect of moderate-intensity physical exercise over 6 months is the best. In addition, Hayden and Allen (1984) conducted a controlled experimental study on the relationship between aerobic exercise and individual depression and found that individuals who regularly participate in physical exercise for a long time have lower levels of anxiety and depression than non-participants [20]. Yeruva(2019) [21] compared efficacy of four kinds of exercise, steady state aerobic training, resistance training, high intensity interval training(HIIT) and low intensity training. The result showed that most people can benefit from HIIT because it combines the intensity, muscle exhaustion, and testosterone production of resistance training with the cardiovascular advantages of aerobic activities. Aerobic exercise tended to be more effective than resistance training in aiding individuals with depression. It can be found that although there are differences in the selected surveys and researches in the existing literature, they basically believe that physical exercise has a positive effect on mental health.

The relationship between physical exercise and depression has been studied mainly through controlled experiments, questionnaire surveys [22,23], long-term tracking, and Meta-analysis, and there are some differences in research conclusions. Some scholars, Xu et al.(2003), He and Ji(2004) conducted grouped control experiments and found that after physical exercise, the depression level of students decreased significantly [24,25], but the degree of depression improvement was affected by sports events and duration. Influence, there are individual differences [25]. Tang and Zhang(2008) further studied the mechanism of the effect of physical exercise on the degree of depression [26], that is, physical exercise helps to vent bad emotions, gain sports pleasure, and establish good interpersonal relationships. But he also found that when the exercise intensity is too high, physical exercise will make the body fatigue, and may also cause sports injuries, making individuals fear exercise.

Another group of scholars mainly use large-scale micro-survey data to explore the relationship between the two. For example, Baker et al. (2005) used the CESD depression score and healthy exercise indicators in the Americans’ Changing Lives (ACL) panel survey to test the results through OLS and the ordered logistic regression model [11]. The relationship between participation in outdoor activities and mental health of adults over 60 years old has found that increasing the frequency and time of physical exercise can alleviate depression. Rasciute and Downward (2010) used the survey data of 28,117 children over 16 years of age conducted by the UK Market Research Agency in 2005 to demonstrate this point [13]. Santos et al. (2022) obtained 29,285 individuals data from the European Social Survey (ESS) [27], which included 20 European countries (i.e., Austria, Belgium, Czech Republic, Denmark, Estonia, Finland, France, Germany, Hungary, Ireland, Lithuania, Netherlands, Norway, Poland, Portugal, Slovenia, Spain, Sweden, Switzerland, and United Kingdom). They found that more time spent in TV watching is related with increased scores on depressive symptoms and regular physical activity participation can weaken this association.

However, some scholars have found that physical exercise may not have an impact on depression, or the effect is minimal. Scholars such as Fu and Zhou(2004) surveyed 140 students in a university in Hubei and found that there was no significant difference in depression levels among college students with different physical exercise levels [28]. Similarly, Birkeland et al. (2009) used the Norwegian Long-Term Healthy Behavior Survey (NLHB) 10-year survey data of 1195 children and their parents and found that there may be no causal relationship between leisure sports activities and depression [12]. However, the disadvantage of the above-mentioned research is that the two articles did not control other personal and family characteristics that affect the level of depression, so the results may have certain deviations, and other factors need to be further controlled to be able to draw credible conclusions.

In early 2020, when the Covid-19 broke out, governments around the world issued curfews and lockdown, people could not do outdoor activities as usual and have to use digital technologies such as Internet, social media, smartphones, more than ever before [29]. Many scholars regarded this as a natural experiment, using questionnaires or social media comments to study public emotions and mental depression. This provides new evidence for our study of the relationship between physical activity and depression. The Covid-19 undoubtedly affected people’s mental health. Arora et al. (2021) harvested posts made on Twitter by Indian individuals expressing their feeling or viewpoint on the epidemic during the first 40 days of the shutdown [30]. Users that tweeted more frequently were generally furious, outraged, or depressed about the current situation. Mary-Krause et al. (2021) used generalized estimation equation (GEE) models to study the relationship between COVID-19-like symptoms and anxiety/depression [31]. The authors collected longitudinal data among 729 persons in the context of the French TEMPO cohort between March and June 2020 During lockdown, and found that 27.2 percent of research participants experienced anxiety or sadness. During the duration of the study, 17.1 percent of patients had COVID-19-like symptoms, 7.3 percent following the start of lockdown, with an average of 2.7 symptoms, and 3.6 percent suffered respiratory distress. Woon et al. (2021) studied the severity of depressive, anxiety, and stress symptoms among 316 university students, as well as the relationship between various factors and levels of depressive and anxiety symptoms in response to the COVID-19 pandemic after the movement control order (MCO) was lifted. The authors discovered that 15.5%, 11.7%, and 9.2% of the subjects experienced mild, moderate, or severe to extremely severe depression, respectively. According to the multiple linear regression model, annoyance due to loss of daily routine and study disturbance were related with higher depressed symptoms [32].

## Econometric model and data

### Data

Different from existing research, this article uses the 2016 China Family Panel Studies (CFPS) data released by the Chinese Social Science Survey Center of Peking University, which covers a wider range and has a stronger sample representation. While, CFPS has already launched the 2020 survey, but this part of the data has not been made public (it may only be disclosed to Peking University), so it is difficult for us to obtain authorization for data use. In addition, because we studied the effect of physical activity on depression, the time limit of this study is not obvious. Therefore, the update of the data will not have a great impact on our conclusions. Finally, due to the outbreak of COVID-19 worldwide in 2020, including China. The Chinese government’s lockdown policy has left many residents indoors for extended periods of time, which can negatively impact people’s mental health [33,34]. Using pre-pandemic data can also effectively avoid this potential disruption. At the same time, in order to increase the representativeness of the research, this article selects the adult group data, this group covers samples over 16-year-old and provides detailed individual characteristics, physical exercise status, and depression scale scores.

#### Descriptive statistics

Table 1 lists the descriptive statistics of the variables included in the basic model. It can be seen that the average depression score of the individuals in the sample is 32.58 points (SD = 8.06), the average weekly exercise frequency is 2.16 (SD = 3.05), and the average age of the studied individuals is 45.77 years old (SD = 16.92).

**Table 1:** Descriptive statistics (overall, no-sport and sports sample).

Variables	Overall			No-sport	Sport	Mean Difference
	Obs	Mean	Sd	N = 19372	N = 13864	
depression	33236	32.58	8.06	33.03	31.94	1.09***
sport_freq	33236	2.16	3.05	0	5.18	-5.18***
age	33236	45.77	16.92	45.73	45.82	-0.09
edu	33236	2.70	1.39	2.46	3.03	-0.57***
marriage	33236	2.07	0.84	2.10	2.03	0.07***
healthy	33236	2.97	1.22	2.93	3.04	-0.11***
familysize	33236	4.30	2.03	4.47	4.04	0.426***

Note: According to whether the number of exercises is 0, the mean difference test is performed

*** means 1% (two-tailed) statistical significance level.

*p < 0.1; **p < 0.05; ***p < 0.01.

Data source: Authors’ calculations.

It should be noted that the education level in this article is divided into “illiterate/semi-literate, elementary school, junior high school, high school, college, university, master’s degree, doctorate” according to different grades, with values of 1–8 respectively; while marital status is divided into 5 types, That is, "divorced, widowed, cohabiting, unmarried, married" are assigned a value of 1–5; health status is assigned a value of 1–5 according to "unhealthy, normal, relatively healthy, healthy, and very healthy". In addition, according to whether the number of exercises is 0, we divides the sample into a non-exercise group and an exercise group and conducts a mean difference test of the main variables. The results showed that the depression score of the exercise group was 1.09 points lower than that of the non-exercise group, and it was significant at the 1% level. Preliminary analysis can be considered that physical exercise has a negative effect on depression levels. Except for age, the test results of other control variables are also very significant, indicating that the selection of control variables in this article is reasonable.

### Variables and empirical models

According to the research topics and the availability of data, referring to previous studies, this paper constructs the following multivariate linear model

$${Depression}_{i}=\alpha +\beta {sports\_freq}_{i}+\sum \gamma_{k}{person\_control}_{ki}+\delta {province}_{i}+\varphi {month}_{i}+\varepsilon_{i}$$ (1)

Among them, *Depression* is the dependent variable, which represents the depression degree score of individual i. The higher the score, the higher the depression level. This indicator uses the CES-D20 (Center for Epidemiologic Studies Depression Scale) test scores of individuals from 2016 CFPS data, this version contains twenty questions (example: “*how often they felt depressed in the last week*”). Each item is scored from 1 (none or almost none of the time) to 4 (all or almost all the time) with a range between 0 to 60, four items were reverse coded. CES-D20 is a valid and reliable instrument for measuring depression in several studies. *α* is a constant term, *sports_freq* represents the number of exercises per week of individual i. Sport activity is derived from respondents’ responses to "How many times have you exercised in the past week", with more times representing more physical activity. According to the definition of CFPS2016, exercise includes walking, long-distance running, jogging and mountain climbing, etc., practicing Tai Chi and other martial arts and qigong exercises, indoor and outdoor dancing, aerobics, yoga, etc., various ball sports such as big and small balls, swimming, etc., diving, rowing, sailing and other water sports, winter ice, snow sports and physical contact sports such as wrestling, judo, boxing, etc. *person_control* is a series of control variables for individual character including: *age* represents the individual’s age; *edu* represents the individual’s education level; *gender* represents the individual’s gender. *marriage* is a variable that represents the individual’s marital status, and *healthy* represents the self-evaluated health of the individual. In our study, the health variable came from the question "How do you think your health is" in the CFPS2016 survey. Investigators were asked to assess their own health status, choosing among "unhealthy, average, relatively healthy, quite healthy and very healthy". These options are assigned a scale of 1–5, with higher scores indicating better fitness. *familysize* represents the size of the individual’s family. *Province* is the province where the individual is located, *month* represents the month when the individual was surveyed. Both are used to control the location and time fixed effects that cannot be measured, and *ε* is the error term.

## Result and discussion

The empirical works are arranged as follows: first, perform a benchmark model regression; second, use propensity score matching (PSM) estimation method to solve the endogeneity problem; finally, conduct robustness test by transforming the core explanatory variables and heterogeneity analysis by dividing sample into several sub-groups.

### Benchmark regression

Table 2 presents our primary results, with the first two columns contain only independent variable *sports_freq*, column (3) and (4) add control variables of individual characteristic, column (5) and (6) include family characteristic variables. The difference between odd-numbered columns and even-numbered columns is that the odd-numbered columns estimate variable coefficients using the ordinary least squares (OLS) method, whereas the even-numbered columns use two-way fixed effect (TWFE), which controls both province and month fixed effects. Thus we derives the following 6 regression models.

**Table 2:** Multiple regression estimation results of physical exercise on depression level.

	(1)	(2)	(3)	(4)	(5)	(6)
	OLS	TWFE	OLS	TWFE	OLS	TWFE
sports_freq	-0.152***	-0.137***	-0.085***	-0.089***	-0.091***	-0.096***
	(-10.52)	(-9.51)	(-6.21)	(-6.58)	(-6.63)	(-7.02)
Age			-0.059***	-0.045***	-0.062***	-0.048***
			(-18.58)	(-13.97)	(-19.45)	(-14.93)
Edu			-0.713***	-0.633***	-0.748***	-0.655***
			(-21.32)	(-18.38)	(-22.15)	(-18.98)
Gender			-0.709***	-0.801***	-0.714***	-0.810***
			(-8.51)	(-9.71)	(-8.58)	(-9.83)
Marriage			1.004***	0.961***	0.999***	0.958***
			(18.06)	(17.48)	(17.97)	(17.44)
Healthy			-2.260***	-2.238***	-2.255***	-2.228***
			(-62.50)	(-62.33)	(-62.37)	(-62.06)
familysize					-0.148***	-0.173***
					(-7.18)	(-8.02)
_cons	32.907***	32.116***	42.386***	43.844***	43.279***	44.366***
	(608.52)	(5.71)	(174.69)	(8.42)	(158.82)	(8.53)
Province FE	N	Y	N	Y	N	Y
Month FE	N	Y	N	Y	N	Y
N	33236	33236	33165	33165	33165	33165
R-sqaure	0.003	0.030	0.148	0.172	0.151	0.174

Note: T-statistics for each coefficient are in parentheses.

*, **, and *** indicate 10%, 5%, and 1% significance levels, respectively.

Data source: Authors’ calculations.

It can be seen from Table 2 (1) that the regression coefficient of physical exercise frequency is about -0.152, and it is significant at the 1% level. This shows that there is a significant negative correlation between the frequency of physical exercise and the level of depression. For every increase in physical exercise, the level of depression decreases by 0.152 points. In addition, it can be found that with the inclusion of individual characteristic variables and family characteristic variables, the regression coefficients of physical exercise are still significantly negative, and both are significant at the 1% level, and there is no change. The results are very robust. In addition, it is worth noting that the depression level of men is significantly lower than that of women by 0.810 points (taking Model 6 as an example), which is consistent with the conclusions of clinical studies(26).

### Endogenous treatment

Refer to Waldman, Michael & Nicholson, Sean & Adilov, Nodir [15], considering that physical exercise may have an endogenous problem with depression, that is, people who regularly participate in physical exercise are often groups with low levels of depression, And depressed groups often do not participate in physical exercise. In order to solve the possible impact of this "selection bias" on research. The article uses Propensity Score Matching to estimate the "processing effect" of physical exercise on the level of depression. The main idea of this method is: suppose that individual i belongs to the treatment group, and find a certain body j belonging to the control group, so that the measurable variables of the individual i and the individual j are as similar as possible. Then we use control group’s individual j as the initial value of the treatment group individual i before treatment, to estimate the size of the treatment effect. Before the score matching estimation, the balance test is first performed in Table 3.

**Table 3:** Balance test results.

Variables	Treatment	Mean		Error ratio	Error reduction ratio	T-test	
		Treatment Group	Control Group			t	P>t
age	Before	45.841	45.761	0.5		0.43	0.669
	After	45.842	45.521	1.9	-298.1	1.56	0.120
edu	Before	3.0253	2.4591	41.2		37.33	0.000***
	After	3.0250	3.0260	-0.1	99.8	-0.06	0.954
gender	Before	0.5224	0.4825	8.0		7.17	0.000***
	After	0.5223	0.5160	1.3	84.2	1.05	0.295
marriage	Before	2.0251	2.0952	-8.3		-7.46	0.000***
	After	2.0252	2.0076	2.1	74.8	1.82	0.069
healthy	Before	3.0342	2.9279	8.8		7.83	0.000***
	After	3.0340	3.0264	0.6	92.9	0.52	0.602
familysize	Before	4.0471	4.4724	-21.2		-18.89	0.000***
	After	4.0470	4.0401	0.3	98.4	0.30	0.761

Note: Due to space limitations, only a one-to-one matching balance test is reported.

*, **, and *** indicate 10%, 5%, and 1% significance levels, respectively.

Data source: Authors’ calculations.

The test results show that the standardized error ratios of all covariates after matching are less than 10%, and after matching, the t-test results show that there is no significant difference between the two groups. This shows that after propensity score matching, the differences in individual characteristics between the exercise group and the non-exercise group have been eliminated to a greater extent.

In Table 4, models (1)-(4) respectively report the effects of physical exercise on depression levels estimated using one-to-one matching, caliper matching, spline matching, and K-nearest neighbor matching (K = 4) methods. Among them, ATE represents the matching estimation results of all samples; ATU means that only the sample matching estimation results of the non-sports group are considered; ATT is the average processing effect of the samples in the sports group. Table 5 shows that all the matching results are significantly negative at the 1% level, indicating that participating in physical exercise reduces the level of depression by about 0.368 points (using Model 1) as an example.

**Table 4:** Endogenous test results.

Depression	(1)	(2)	(3)	(4)
Matching method	1:1 nearest neighbor	Caliper matching	Spline matching	K nearest neighbor matching
ATT	-0.518***	-0.452***	-0.465***	-0.454***
	(0.138)	(0.115)	(0.090)	(0.109)
ATU	-0.260**	-0.322**	-0.270***	-0.320***
	(0.129)	(0.132)	(0.099)	(0.114)
ATE	-0.368***	-0.376***	-0.351***	-0.376***
	(0.100)	(0.107)	(0.091)	(0.095)
N	33,156	33,156	33,156	33,156

Note: (1)-(4) represent different matching methods, and the standard deviations reported in the above table are all obtained through the bootstrap method.

*, **, and *** indicate 10%, 5%, and 1% significance levels, respectively.

Data source: Authors’ calculations.

**Table 5:** Robustness test results.

	Depression		
	(1)	(2)	(3)
sport		-0.397***	
		(-4.69)	
sports_freq	-0.102***		
	(-7.44)		
sport_time			-0.033***
			(-6.19)
sport_time2			0.001***
			(4.61)
age	-0.051***	-0.051***	-0.050***
	(-14.51)	(-15.83)	(-15.44)
edu	-0.656***	-0.657***	-0.651***
	(-18.85)	(-18.87)	(-18.77)
gender	-0.745***	-0.809***	-0.807***
	(-8.90)	(-9.82)	(-9.80)
marriage	0.943***	0.961***	0.958***
	(17.12)	(17.48)	(17.42)
healthy	-2.216***	-2.231***	-2.228***
	(-61.59)	(-62.15)	(-62.04)
jobless	0.444***		
	(4.57)		
income	0.000		
	(0.99)		
familysize	-0.173***	-0.170***	-0.173***
	(-8.04)	(-7.87)	(-8.01)
child	0.003		
	(0.12)		
_cons	44.366***	44.983***	44.605***
	(8.53)	(8.65)	(8.58)
Province FE	Y	Y	Y
Month FE	Y	Y	Y
N	33165	33157	33165
R-Square	0.174	0.173	0.173

Note: T-statistics for each coefficient are in parentheses.

*, **, and *** indicate 10%, 5%, and 1% significance levels, respectively.

Data source: Authors’ calculations.

### Robustness test

We adopt three methods to ensure the robustness of our conclusions. First, we add three control variables to exclude factors that might influence physical exercise and depression level simultaneously, which are unemployment status (*jobless*) and income level (*income*) as individual characteristics, and children (*child*) at household characteristic. Second, we replace the key explanatory variables *sport**_**freq* with *sport*, a dummy variable of whether the individual participates in physical exercise. Third, we substitute *sport**_**freq* with *sport**_**time*, the duration of physical exercise to analyze the relationship between duration of physical exercise and depression. The results are shown in Table 5.

The results in Table 5 show that after adding three control variables, the anti-depression effect of physical exercise is still significant at 1% level. Specifically, the depression level of jobless people is 44.4% higher than those who are employed while income and the number of children have no significant influence on people’s depression level. When explanatory variable *sport_freq* is replaced by dummy variable *sport*, the negative correlation between the physical exercise and the level of depression is still established and significant at the 1% level. In addition, it can be found that compared with the benchmark regression analysis, those who participated in sports had a lower level of depression than those who did not participate by 0.397 points. If explanatory variable *sport_freq* is replaced by *sport_time* and its quadratic form, we can find that the relationship between physical exercise and depression is U-shaped instead of linear, which implies that in some extent more physical exercise do bring down depression level but excessive physical exercise might act adversely, namely increase people’s depression level.

In order to ensure the credibility of the research as much as possible,we further add to the discussion of the robustness of the conclusions. First, psychotherapy has a relieving effect on depression, so it needs robust means of managing this concern. Due to data limitations, we do not have direct information about individuals receiving psychotherapy. But in CFPS2016, which provides information on total annual medical expenditures, we narrowed the sample to those with zero total medical expenditures (meaning that these people did not pay for psychotherapy). Second, we considered the quality of the survey. Since the survey is carried out in the form of a questionnaire, the attitude of the respondents towards the survey directly affects the quality of the data. We further narrowed the study sample based on the degree to which respondents cooperated with the survey, the investigator’s judgment of the credibility of the questionnaire, and the degree to which respondents were eager to close the survey. Third, we controlled for the effect of the unfair treatment respondents faced on depression. China has a huge population base, and complex social relations may lead to unfair treatment of some people for various reasons. Such as facing barriers in employment because of gender, discrimination because of household registration, and poor communication with government officials. We control for injustices suffered by individuals based on gender, wealth, household registration, and government officials. Finally, we considered the effect of religious beliefs on depression. Wink et al. (2005) found that religious belief buffered depression associated with poor physical health [35].

Table 6 presents the results excluding psychotherapy and considering the quality of the questionnaire. The results in column 1 of Table 6 indicate that even in the sample without psychotherapy, physical exercise still had a significant inhibitory effect on depression. The last three columns of Table 6 consider the impact of the respondent’s degree of cooperation, credibility, and eagerness to end the survey on the quality of the questionnaire. We found that the results remain robust. The first four columns of Table 7 include the discrimination suffered by individuals based on gender, wealth, household registration, and politics, respectively. For the above four variables, the larger the value, the smaller the unfair treatment suffered. We found higher levels of depression when treated unfairly. More importantly, the effect of physical activity on depression was still significantly negative. The last two columns of Table 7 consider the effect of religious belief on depression. We found that worship has an inhibitory effect on depression and Buddhism on depression, and religious belief helps distract individuals, thereby reducing psychological barriers. More importantly, after the above robustness test, the reducing effect of physical exercise on depression still holds.

**Table 6:** Psychotherapy and questionnaire quality.

	(1)	(2)	(3)	(4)
	Exclude psychotherapy	Questionnaire quality		
sports_freq	-0.097***	-0.108***	-0.103***	-0.125***
	(-4.22)	(-6.80)	(-6.42)	(-6.61)
Age	-0.054***	-0.059***	-0.060***	-0.058***
	(-9.95)	(-15.29)	(-15.52)	(-12.70)
Edu	-0.518***	-0.665***	-0.627***	-0.746***
	(-9.38)	(-16.70)	(-15.77)	(-15.33)
Gender	-0.489***	-0.891***	-0.804***	-0.826***
	(-3.68)	(-9.21)	(-8.24)	(-7.13)
Marriage	0.852***	0.968***	0.952***	1.000***
	(8.47)	(14.33)	(13.54)	(12.73)
Healthy	-1.678***	-2.228***	-2.191***	-2.213***
	(-26.92)	(-52.33)	(-50.45)	(-44.13)
Familysize	-0.177***	-0.139***	-0.113***	-0.150***
	(-5.05)	(-5.28)	(-4.20)	(-4.78)
_cons	51.961***	44.667***	44.219***	44.923***
	(11.03)	(8.71)	(8.77)	(8.54)
Province FE	Y	Y	Y	Y
Month FE	Y	Y	Y	Y
N	10419	23220	21979	17066
R-sqaure	0.118	0.172	0.165	0.173

Note: T-statistics for each coefficient are in parentheses.

*, **, and *** indicate 10%, 5%, and 1% significance levels, respectively.

Data source: Authors’ calculations.

**Table 7:** Unfair treatment and religion.

	(1)	(2)	(3)	(4)	(5)	(6)
	gender injustice	wealth injustice	registration injustice	government officials	Buddha	worship
sports_freq	-0.087***	-0.089***	-0.087***	-0.089***	-0.095***	-0.096***
	(-6.55)	(-6.63)	(-6.43)	(-6.63)	(-7.00)	(-7.07)
Age	-0.045***	-0.047***	-0.048***	-0.049***	-0.048***	-0.048***
	(-14.05)	(-14.74)	(-15.01)	(-15.43)	(-14.93)	(-14.89)
Edu	-0.568***	-0.610***	-0.620***	-0.590***	-0.655***	-0.654***
	(-16.86)	(-17.92)	(-18.22)	(-17.35)	(-18.99)	(-18.94)
Gender	-1.013***	-0.879***	-0.691***	-0.966***	-0.812***	-0.804***
	(-12.55)	(-10.79)	(-8.47)	(-11.87)	(-9.86)	(-9.75)
Marriage	0.867***	0.911***	0.943***	0.921***	0.960***	0.955***
	(15.94)	(16.54)	(17.15)	(16.90)	(17.46)	(17.37)
Healthy	-2.064***	-2.142***	-2.167***	-2.139***	-2.228***	-2.227***
	(-58.39)	(-60.06)	(-60.86)	(-60.23)	(-62.07)	(-62.06)
Familysize	-0.163***	-0.158***	-0.156***	-0.163***	-0.173***	-0.172***
	(-7.73)	(-7.42)	(-7.33)	(-7.66)	(-8.02)	(-8.00)
_cons	47.280***	47.630***	51.284***	48.931***	44.696***	44.673***
	(9.40)	(9.36)	(10.03)	(9.58)	(8.59)	(8.59)
gender injustice	-1.219***					
	(-42.37)					
wealth injustice		-1.224***				
		(-29.80)				
registration			-1.469***			
Injustice			(-26.31)			
government officials injustice				-0.962***		
				(-30.22)		
Buddha					-0.019	
					(-1.08)	
Worship						-0.030**
						(-2.15)
Province FE	Y	Y	Y	Y	Y	Y
Month FE	Y	Y	Y	Y	Y	Y
N	32445	32469	32635	32938	33165	33165
R-sqaure	0.214	0.191	0.187	0.194	0.174	0.174

Note: T-statistics for each coefficient are in parentheses.

*, **, and *** indicate 10%, 5%, and 1% significance levels, respectively.

Data source: Authors’ calculations.

### Heterogeneity analysis

CFPS 2016 survey contains more than 30,000 individuals over the age of 16 and in order to explore the effect among different age groups, we divide the sample into six sub-groups by age under 36 years old, 36–45 years old, 46–55 years old, 56–65 years old, 66–75 years and over 76 years old. The results are shown in Table 8.

**Table 8:** Heterogeneity analysis of different age.

	(1)	(2)	(3)	(4)	(5)	(6)
	Under 36	36–45	46–55	56–65	66–75	Above 76
sports_freq	-0.019	-0.024	-0.042	-0.114***	-0.187***	-0.373***
	(-0.82)	(-0.65)	(-1.36)	(-3.42)	(-4.56)	(-5.29)
Age	0.040***	-0.079**	-0.003	-0.141***	-0.012	0.002
	(2.71)	(-2.29)	(-0.09)	(-3.36)	(-0.23)	(0.02)
Edu	-0.586***	-0.710***	-0.716***	-0.858***	-1.114***	-0.549**
	(-11.88)	(-8.62)	(-8.55)	(-8.23)	(-7.43)	(-2.36)
Gender	-0.456***	-0.441**	-1.129***	-1.295***	-0.967***	-1.097**
	(-3.69)	(-2.26)	(-6.00)	(-5.66)	(-3.23)	(-1.98)
Marriage	0.184	1.102***	1.264***	1.134***	1.027***	0.301
	(1.35)	(5.38)	(8.32)	(8.46)	(8.22)	(1.63)
Healthy	-1.729***	-2.146***	-2.245***	-2.554***	-2.610***	-2.664***
	(-28.43)	(-25.32)	(-29.37)	(-28.25)	(-21.13)	(-12.09)
Familysize	-0.107***	0.014	-0.200***	-0.266***	-0.332***	-0.252**
	(-3.28)	(0.23)	(-3.71)	(-4.83)	(-4.68)	(-2.03)
_cons	40.163***	47.918***	53.705***	84.835***	50.790***	37.658***
	(8.99)	(10.76)	(6.83)	(10.09)	(5.64)	(5.57)
Province FE	Y	Y	Y	Y	Y	Y
Month FE	Y	Y	Y	Y	Y	Y
N	10726	5411	6982	5392	3424	1230
R-Square	0.121	0.166	0.184	0.220	0.241	0.228

Note: T-statistics for each coefficient are in parentheses.

*, **, and *** indicate 10%, 5%, and 1% significance levels, respectively.

Data source: Authors’ calculations.

It can be found in Table 8 that, under the control of the province, month effect and other variables, the effect of physical exercise frequency on depression level increased from -0.019 points to -0.373 points as the age group increased. Moreover, in the group under 55, the effect of physical exercise is not significant. But in the 56-year-old group, its influence tends to expand. For those over the age of 76, the depression level decreased by 0.373 points for every increase in physical exercise. This shows that people over 76-year-old should increase physical exercise appropriately to reduce the occurrence of psychological problems such as loneliness and depression.

Secondly, we take into account the differences in individual education level. After regressing the individual’s education level by group we obtain the estimated results in Table 9. It can be found that the effect of physical exercise on the level of depression is greatest in the primary school and college/university level groups. For every additional time of physical exercise, the depression level of the group with elementary education level decreased by 0.124 points, while the level of depression in the university/college group decreased by 0.116 points. It is worth noting that the impact effect of illiterate/semi-literate also reached 0.106 points, which may be caused by groups with low academic qualifications who may not have more abundant ways to relieve life and psychological stress in their spare time. Physical exercise is an important way for this group to prevent mental health problems.

**Table 9:** Heterogeneity analysis of different education level.

	(1)	(2)	(3)	(4)	(5)	(6)
	Illiteracy/ Half-illiteracy	Primary School	Junior High School	High School	University/College	Above Master
sports_freq	-0.106***	-0.124***	-0.051**	-0.060*	-0.116***	-0.471
	(-3.41)	(-4.02)	(-2.16)	(-1.91)	(-2.85)	(-1.44)
Age	-0.025***	-0.049***	-0.060***	-0.063***	-0.043***	-0.015
	(-3.20)	(-6.94)	(-10.42)	(-8.23)	(-3.82)	(-0.13)
Gender	-1.398***	-0.489***	-0.552***	-0.692***	-0.388*	0.205
	(-7.15)	(-2.61)	(-3.83)	(-3.61)	(-1.78)	(0.13)
Marriage	0.862***	1.018***	1.058***	1.031***	0.268	0.098
	(9.19)	(7.74)	(9.03)	(6.74)	(1.33)	(0.06)
Healthy	-2.424***	-2.248***	-2.033***	-2.156***	-1.924***	-1.345
	(-32.88)	(-28.67)	(-31.40)	(-23.05)	(-16.86)	(-1.38)
Familysize	-0.345***	-0.158***	-0.043	-0.113**	0.009	0.046
	(-7.70)	(-3.19)	(-1.13)	(-2.13)	(0.13)	(0.07)
_cons	56.212***	58.558***	43.454***	39.049***	43.271***	27.633***
	(9.38)	(13.16)	(6.31)	(5.92)	(6.88)	(3.03)
Province FE	Y	Y	Y	Y	Y	Y
Month FE	Y	Y	Y	Y	Y	Y
N	8356	6844	9565	4893	3389	118
R-Square	0.187	0.159	0.131	0.141	0.118	0.228

Note: T-statistics for each coefficient are in parentheses.

*, **, and *** indicate 10%, 5%, and 1% significance levels, respectively.

Data source: Authors’ calculations.

Thirdly, we attempt to figure out the heterogeneous effect of physical exercise on people with high/low level of depression. As there is no universal cut-off value in CES-D20 to identify depression symptoms, we applied median level of depression as a threshold and derived two sub-group. The estimated results are concluded in Table 10. It can be observed that physical exercise can reduce depression level in both groups, for each additional physical exercise, the sample with low depression levels decreased symptoms by 0.025, while the sample with high depression levels decreased symptoms by 0.041. Therefore, physical exercise as a way to decrease depression symptom is more effective among high level depression people.

**Table 10:** Heterogeneity analysis of high/low depression.

	(1)	(2)
	low depression	high depression
sport_freq	-0.025***	-0.041**
	(-3.51)	(-2.47)
Age	-0.022***	0.003
	(-12.80)	(0.79)
Edu	-0.102***	-0.502***
	(-5.69)	(-11.86)
Gender	-0.178***	-0.292***
	(-4.14)	(-2.94)
Marriage	0.090***	0.578***
	(2.66)	(9.79)
Healthy	-0.387***	-1.340***
	(-19.39)	(-30.49)
Familysize	0.010	-0.186***
	(0.91)	(-7.21)
_cons	29.377***	41.182***
	(10.86)	(6.54)
Province FE	Y	Y
Month FE	Y	Y
N	16310	16855
R-Square	0.046	0.120

Note: T-statistics for each coefficient are in parentheses.

*, **, and *** indicate 10%, 5%, and 1% significance levels, respectively.

Data source: Authors’ calculations.

### Extension

Different kinds of physical exercise might have distinct influence on depression level. But there is no granular data on sports types in CFPS 2016. Therefore, we have to transfer to another survey data, China Health and Nutrition Survey (CHNS). CHNS is jointly released by the University of North Carolina and the Chinese Center for Disease Control and Prevention, which is conducted by an international team of researchers with professional backgrounds in nutrition, public health, economics, sociology, China studies and demography. The survey was conducted over a period of 7 days, using a multi-stage, random clustering method, involving residents of 12 provinces across the country. Questions are on demographic background, work and income, housework activities, tobacco and alcohol consumption, physical activity, diet, disease, marriage, etc. Due to the large amount of data and rich information, it has been used in most studies.

Since questions on sports types were added since 2015, we use CHNS from 2015 in our extension analysis, after excluding missing values we get a sample of 5570. Different from CFPS 2016, CES-D20 was not used in CHNS to evaluate individual depression, while there is a similar part (P26) on pressure in CHNS, which contains fourteen questions (example: “*Are you nervous and stressed in the last month*?”) with each item scored from 1 (never) to 5 (always). From those questions, we constructed a proxy variable of depression level from CHNS. We used the respondents’ answers to the survey on six sports categories, gymnastics (*gym*), athletics and swimming (*ath*), walking (*walk*), football, basketball and tennis (*fbt*), badminton and volleyball (*bv*), other activities (*others*) to respectively constructed continuous variables for regression and got the results in Table 11. To illustrate that the two sets of data have similar structures, we performed a baseline regression of exercise duration and depression level in the first column, and the results showed that in the CHNS data, the negative relationship between exercise and depression level still exists. It can be clearly seen from the table below that *gym*, *ath*, *fbt* and *others* have no significant effect on reducing depression level, while *walk* and *bv* are significant at 1% level. Specifically, walking reduced the level of depression by 0.005, badminton and volleyball reduced the level of depression by 0.027.

**Table 11:** The effect of different physical exercise.

	Depression						
	(1)	(2)	(3)	(4)	(5)	(6)	(7)
sport_time	-0.004***						
	(-3.41)						
age	-0.026***	-0.025	0.003	-0.027***	-0.045	-0.038	-0.033
	(-4.20)	(-0.94)	(0.11)	(-4.17)	(-1.03)	(-0.90)	(-1.30)
edu_year	0.035***	-0.007	0.013	0.033***	-0.044	0.061	-0.143**
	(2.97)	(-0.14)	(0.20)	(2.67)	(-0.56)	(0.58)	(-2.48)
gender	-0.060	-1.254	-0.671	-0.081	-3.576	0.253	-0.804
	(-0.38)	(-1.03)	(-0.82)	(-0.49)	(-1.17)	(0.28)	(-0.87)
marry	0.349**	0.208	0.325	0.334*	0.601	0.729*	0.033
	(2.39)	(0.69)	(0.66)	(1.84)	(0.56)	(1.90)	(0.13)
health	0.186	0.784	0.013	0.261	0.420	0.458*	0.363
	(0.90)	(0.86)	(0.01)	(1.19)	(1.59)	(1.68)	(0.40)
gym		0.004					
		(1.41)					
ath			-0.006				
			(-1.20)				
walk				-0.005***			
				(-3.44)			
fbt					0.002		
					(0.26)		
bv						-0.027***	
						(-3.60)	
others							-0.006
							(-1.31)
_cons	26.024***	30.089***	24.435***	26.324***	30.094***	24.103***	32.517***
	(35.38)	(7.81)	(6.37)	(32.62)	(7.40)	(5.57)	(9.77)
Province FE	Y	Y	Y	Y	Y	Y	Y
Month FE	Y	Y	Y	Y	Y	Y	Y
N	5570	425	238	5006	155	165	223
r2	0.024	0.047	0.084	0.025	0.159	0.136	0.059

Note: T-statistics for each coefficient are in parentheses.

*, **, and *** indicate 10%, 5%, and 1% significance levels, respectively.

Data source: Authors’ calculations.

Researchers have found that physical activities (PA) have great impact on depression level and in this paper, we only focused on one sub-type of PA, physical exercise. Therefore, to study the relationship between PA and depression, we went back to CFPS 2016, which provided data on five kinds (surfing the internet, doing housework, watching TV/movie, eating with family and reading) of physical activities except for physical exercise. The regression results are presented in Table 12.

**Table 12:** The effect of different physical activities.

	Depression				
	(1)	(2)	(3)	(4)	(5)
Age	-0.049***	-0.052***	-0.049***	-0.042***	-0.053***
	(-14.04)	(-16.29)	(-15.15)	(-13.05)	(-16.43)
Edu	-0.711***	-0.689***	-0.682***	-0.705***	-0.675***
	(-20.10)	(-20.12)	(-19.98)	(-20.68)	(-19.44)
Gender	-0.813***	-0.804***	-0.772***	-0.939***	-0.812***
	(-9.87)	(-9.57)	(-9.38)	(-11.39)	(-9.85)
Marriage	0.967***	0.972***	0.958***	0.839***	0.970***
	(17.59)	(17.69)	(17.47)	(15.16)	(17.65)
Healthy	-2.238***	-2.237***	-2.239***	-2.245***	-2.238***
	(-62.37)	(-62.25)	(-62.53)	(-62.81)	(-62.35)
Familysize	-0.162***	-0.164***	-0.177***	-0.076***	-0.166***
	(-7.50)	(-7.62)	(-8.23)	(-3.46)	(-7.71)
Internet	0.010**				
	(2.54)				
Housework		0.005			
		(0.53)			
Tvmovie			-0.048***		
			(-12.28)		
Familyeat				-0.187***	
				(-16.75)	
Read					-0.007**
					(-2.10)
_cons	44.580***	44.733***	44.618***	43.975***	44.660***
	(8.57)	(8.59)	(8.59)	(8.48)	(8.58)
Province FE	Y	Y	Y	Y	Y
Month FE	Y	Y	Y	Y	Y
N	33165	33165	33165	33165	33165
r2	0.172	0.172	0.176	0.179	0.172

Note: T-statistics for each coefficient are in parentheses.

*, **, and *** indicate 10%, 5%, and 1% significance levels, respectively.

Data source: Authors’ calculations.

What we can learn from the result is that watching TV/movie (-0.048), eating with family members (-0.187) and reading (-0.007) can decrease the level of depression prominently, while doing housework has no significant impact on depression. Surprisingly, we find that surfing the internet may increase the level of depression at 5% statistical significance.

### Discussion

From January 23, 2020, in order to prevent and control the pandemic, Wuhan, the provincial capital city of central China, has been closed down. Not until April 9, people were allowed to leave Wuhan or Hubei Province. During the period of time, universities, restaurants, shopping malls, and communities, not only in Wuhan, but also across the country, have implemented strict blockade measures. The lockdown for more than two months provided us with an excellent opportunity to study physical exercise and depression reduction. The exogenous impact of the epidemic has made people trapped at home and reduced their outings for exercise. The survey data exposed by the latest studies show that people’s depression levels have risen, this indirectly supports the research theme of our research, that is, physical exercise does reduce people’s depression levels.

Sun Zhanning et al.(2020) [36] used an electronic questionnaire in February 2020 to collect the physical exercise status of 2,686 people from multiple age groups in 31 provinces in China. The result showed that 26% of the surveyed people exercise less frequently and the number of people exercising at home has increased. Liang et al.(2021) [37] invited 494 Chinese college students (baseline age 20.41 ± 1.74) from 23 provinces to participate in an online survey in February, May, August, November in 2020 and February in 2021. They found out that the level of depression symptoms increased first and reached the highest in August 2020 and decreased significantly in November 2020. Liu et al. (2021) [38] conducted a follow-up study on 8118 students from 22 colleges and universities in the Guangdong Province of China. Using Patient Health Questionnaire (PHQ-9) to measure depression symptoms, only to find 29% of participants’ depression level increased moderately, 2% are extremely vulnerable during the pandemic.

The above researches prove that the pandemic has indeed reduced exercise time, while at the same time the level of depression has increased. These findings support our conclusions by offering evidence from reality.

## Conclusion and implication

For the first time, this article uses the 2016 China Family Panel Studies (CFPS) data of 33,236 individuals to measure the effect of physical exercise on depression level. Through benchmark regression, it can be found that in China physical exercise reduces the level of depression. The number of physical exercises per week increases once, and the depression level decreases by 0.152 points. However, this relationship is not linear, excessive exercise might lead to higher depression level. In addition, we use three methods to ensure our findings reliable, including adding more control variables, replacing independent variable with the dummy variable and duration of physical exercise, and find that the aforementioned conclusion is still very robust.

Taking into account the endogenous problems caused by selection bias, we applied Propensity Score Matching to investigate the treatment effect of physical exercise. The analysis results found that after solving the endogenous problem, participating in physical exercise can reduce the level of depression by 0.368 points than not participating in physical exercise, and it is significant at the 1% level.

In order to further investigate the physical exercise’s anti-depression effect between different samples, we carry out three heterogeneity tests on age, education and depression level. Among those over 76 years old, one more physical exercise can reduce the level of depression by 0.373 points. In addition, physical exercise has the most significant alleviating effect on the illiterate/semi-literate, elementary school, and college/university groups. Each increase in physical exercise can reduce the depression level of individuals with primary school education by 0.124 points. We also find that physical exercise’s anti-depression effect is more significant on people with high-level depression. Specifically, people with low depression levels add one more physical exercise and their depression levels decreased by 0.025, while people with high depression levels decreased by 0.041. Extensively, we analyzed the effect of six different physical exercises and five different physical activities on the level of depression. This provides a direction for our future research.

Due to space limitations, this article has not been able to conduct an in-depth analysis of the impact mechanism of physical exercise on depression levels in other individual characteristics. However, the ideas, data, and methods provided in this article lay the foundation for the next advancement of research. The research provides theoretical support for the early prevention and late relief of depression patients, and encourages people to actively participate in sports.

## Supporting information

S1 Dataset S1_regdata. (RAR)
